# Automated video-based assessment of facial bradykinesia in de-novo Parkinson’s disease

**DOI:** 10.1038/s41746-022-00642-5

**Published:** 2022-07-18

**Authors:** Michal Novotny, Tereza Tykalova, Hana Ruzickova, Evzen Ruzicka, Petr Dusek, Jan Rusz

**Affiliations:** 1 Department of Circuit Theory, Faculty of Electrical Engineering, Czech Technical University in Prague, Prague, Czech Republic; 2 Department of Neurology and Centre of Clinical Neuroscience, First Faculty of Medicine, Charles University, Prague, Czech Republic; 3 Department of Neurology & ARTORG Center, Inselspital, Bern University Hospital, University of Bern, Bern, Switzerland

**Keywords:** Diagnostic markers, Parkinson's disease

## Abstract

Even though hypomimia is a hallmark of Parkinson’s disease (PD), objective and easily interpretable tools to capture the disruption of spontaneous and deliberate facial movements are lacking. This study aimed to develop a fully automatic video-based hypomimia assessment tool and estimate the prevalence and characteristics of hypomimia in de-novo PD patients with relation to clinical and dopamine transporter imaging markers. For this cross-sectional study, video samples of spontaneous speech were collected from 91 de-novo, drug-naïve PD participants and 75 age and sex-matched healthy controls. Twelve facial markers covering areas of forehead, nose root, eyebrows, eyes, lateral canthal areas, cheeks, mouth, and jaw were used to quantitatively describe facial dynamics. All patients were evaluated using Movement Disorder Society-Unified PD Rating Scale and Dopamine Transporter Single-Photon Emission Computed Tomography. Newly developed automated facial analysis tool enabled high-accuracy discrimination between PD and controls with area under the curve of 0.87. The prevalence of hypomimia in de-novo PD cohort was 57%, mainly associated with dysfunction of mouth and jaw movements, and decreased variability in forehead and nose root wrinkles (*p* < 0.001). Strongest correlation was found between reduction of lower lip movements and nigro-putaminal dopaminergic loss (*r* = 0.32, *p* = 0.002) as well as limb bradykinesia/rigidity scores (*r* = −0.37 *p* < 0.001). Hypomimia represents a frequent, early marker of motor impairment in PD that can be robustly assessed via automatic video-based analysis. Our results support an association between striatal dopaminergic deficit and hypomimia in PD.

## Introduction

The terms characterizing reduction of facial movements such as “masked face” were part of the earliest Parkinson’s disease (PD) descriptions^1,2^. Current research defines facial bradykinesia also known as hypomimia as a loss of spontaneous facial movements and emotional facial expressions, decreased amplitude of the deliberately posed facial expressions, reduced frequency of blinking, and jaw bradykinesia^3^. It has been estimated that up to 92% of all PD patients develop hypomimia in the course of the disease, making it the most common orofacial PD manifestation, followed by speech impairment, dysphagia, and drooling^4^. Hypomimia is also considered to be one of the earliest motor manifestations of PD^4^, developing up to 10 years before the clinical diagnosis of PD, preceding the onset of limb bradykinesia, rigidity gait abnormalities, or resting tremor^5^. However, all these findings^4,5^ are based on a very simple, crude, and routine evaluation of face changes using a subjective 4-point rating scale. To summarize, although being a well-recognized, and common manifestation of PD, the loss of facial expressivity is an understudied topic due to the lack of accessible objective tools for its more precise evaluation^6,7^. Moreover, the objective evaluation of hypomimia in de-novo, drug-naïve PD patients has never been performed, and the potential sensitivity of specific facial markers in early PD remains generally unknown.

This study aims to develop a fully automatic approach based on state-of-the-art computer vision techniques providing a robust, objective, easy to administer, and easy to interpret tool for hypomimia assessment. Based on the proposed approach, we aim to estimate the prevalence and determine quantitative characteristics of hypomimia in a large cohort of de-novo drug-naïve PD patients. An additional purpose is to explore the potential relationships between facial markers and clinical and neuroimaging data to provide greater insight into the pathophysiology of hypomimia in PD.

## Results

### Participants

A total number of 97 PD participants were examined, and six were subsequently excluded: one because the diagnosis was updated to corticobasal degeneration, and five had moderate depression level according to BDI II. As a result, a group of 91 de-novo treatment-naïve PD patients were included in this study consisting of 54 (59%) males and 37 (41%) females with an average age of 61.0 (SD 12.3, range 34–81) years, PD motor symptoms duration 2.0 (SD 1.7) years, and mean MDS-UPDRS part III score 29.8 (SD 12.2) (Table 1). As a healthy control group, 75 white sex- and age-matched participants consisting of 45 (60%) males and 30 (40%) females with an average age of 60.8 (SD 8.8, range 45–86) years were recruited. All PD and control participants were Caucasian and Czech native speakers.

**Table 1:** The list of demographical and clinical information describing PD participants.

Clinical characteristics	PD (*n* = 91; men *n* = 54)
General	
Age (years)	61.0 (12.3, 34–81)
Symptom duration (years)	2.0 (1.7, 0.3–11.3)
Motor manifestations	
MDS-UPDRS III	29.8 (12.2, 6–63)
Bradykinesia/Rigidity	19.9 ± (9.3, 3–46)
PIGD	1.9 ± (1.7, 0–7)
Non-motor manifestations	
MoCA	25.0 ± (3.9, 17–30)
BDI II	7.8 ± (4.7, 0–19)
Brain imaging (DAT-SPECT)	
Caudate binding ratio	3.0 ± (0.6, 1.3–4.3)
Putamen binding ratio	1.5 ± (0.4, 0.9–2.3)

Values are listed in in the format mean (standard deviation, range).

*PD* Parkinson’s disease, *MDS-UPDRS* Movement Disorders Society – Unified Parkinson’s Disease Rating Scale, *PIGD* postural instability/gait difficulty, *MoCA* montreal cognitive assessment, *BDI II* Beck depression inventory II, *DAT-SPECT* dopamine transporter single-photon emission computed tomography.

### Between-group differences

**Fig. 1:**
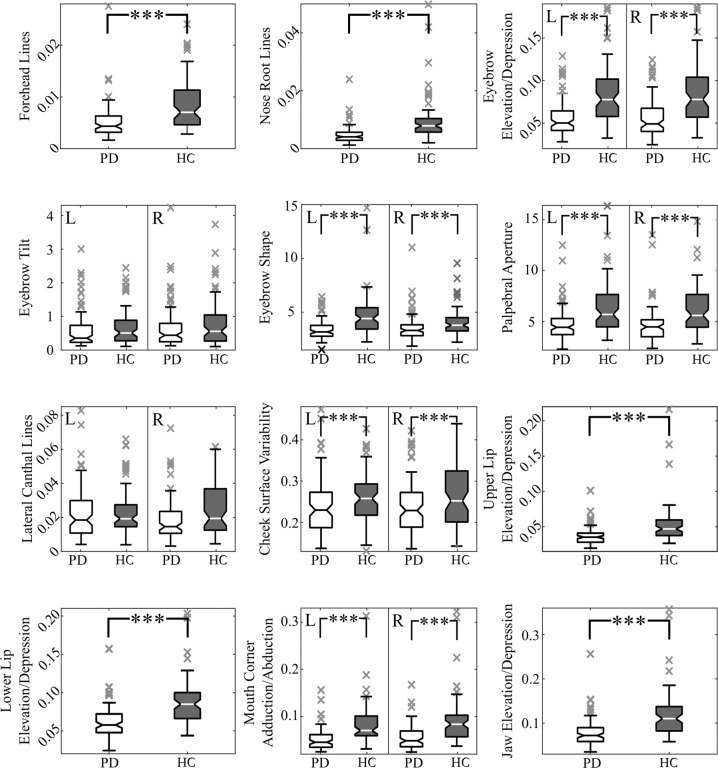
Depiction of between-group differences for each facial marker. The markers with two-sided variant are presented in the same sub-plot and denoted by L for left side and R for the right side. In the figure the centerlines denote feature medians, bounds of boxes represent 25th and 75th percentiles, whiskers denote nonoutlier data range and the crosses denote outlier values. Statistically significant differences after Bonferroni adjustment are denoted by asterisks: ***p < 0.001. PD = Parkinson’s disease; HC = healthy controls.

We revealed significant differences in facial areas, including forehead, nose root, eyebrows (including eyebrow elevation/depression and eyebrow shape), eyes, cheeks, mouth (including upper lip elevation/depression, lower lip elevation/depression, and mouth corner adduction/abduction), and jaw (all analyses significant at *p* < 0.001) (Fig. 1); both left and the right side was significant for those markers involving laterality. Regarding the perceptual assessment, we also found a significant difference (*p* < 0.001) between the average PD group rating 1.3 (SD 1.0, range 0–3) and control group rating 0.3 (0.5, 0–2).

### Comparison of automatic analysis and perceptual assessment

The analysis of relationship between computerized approach (hypomimia is reflected by decrease of facial movements) and perceptual severity assessment (hypomimia is reflected by increase of perceptual score) of merged PD and control datasets revealed significant negative correlations for markers describing facial areas of forehead (*r* = −0.47, *p* < 0.001), nose root (*r* = −0.50, *p* < 0.001), eyebrows (eyebrow elevation/depression: *r* = −0.54, *p* < 0.001; eyebrow shape: *r* = −0.44, *p* < 0.001), eyes (*r* = −0.38, *p* < 0.001), lateral canthal areas (*r* = −0.26, *p* < 0.001), mouth (upper lip elevation/depression: *r* = −0.51, *p* < 0.001; lower lip elevation/depression: *r* = −0.47, *p* < 0.001; mouth corner adduction/abduction: *r* = −0.53, *p* < 0.001), and jaw (*r* = −0.49, *p* < 0.001) (Supplementary Table 1).

### Hypomimia diagnostic sensitivity

**Fig. 2:**
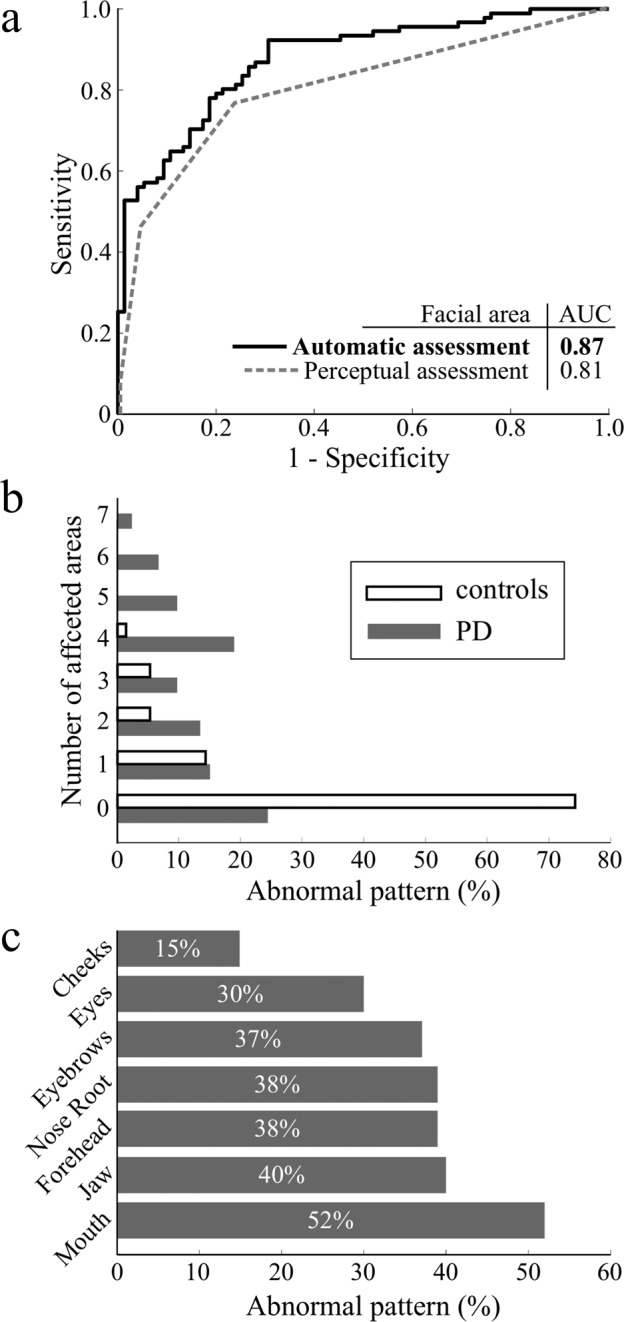
Results of hypomimia sensitivity analysis. (**A**) Receiver operating characteristic curves between PD and controls. The solid line represents automatic assessment with the best overall AUC, which was based on the five facial areas with the best discrimination scores. The dashed line represents the operating characteristic curve based on the 4-point perceptual assessment. **B** Ratios of participants manifesting abnormal patterns in different numbers of affected areas. **C** Ratios of disrupted facial areas in PD.

The different patterns in facial expressivity between PD and control participants led to an overall AUC of 0.87 with an accuracy of 78.3% (sensitivity of 79.1% and specificity of 77.8%) (Fig. 2A); perceptual assessment reached an AUC of 0.81 with the accuracy of 75.9% (sensitivity of 71.6% and specificity 80.0%). The highest overall AUC was achieved by a combination of five markers, including forehead lines (AUC = 0.81), eyebrow elevation/depression (AUC = 0.78), cheek surface variability (AUC = 0.64), mouth corner adduction/abduction (AUC = 0.81), and jaw elevation/depression (AUC = 0.80) (Supplementary Table 2).

Based upon the receiver operating characteristic curve, the prevalence of hypomimia evaluated by automatic video-based assessment in de-novo PD was estimated as 57% while maintaining a false-positive rate under 5%. In comparison, the perceptual assessment evaluated 45% of PD and 4% of control participants as mildly or moderately/severely hypomimic. Considering the agreement of automated and perceptual assessment in PD subset, both methods yielded same result in 50 (55%) of all participants with 26 (29%) subjects classified as hypomimic and 24 (26%) non-hypomimic. The computerized method detected hypomimia in 26 (29%) PD patients whose facial expression was found to be normal by perceptual assessment, whereas hypomimia in 15 (16%) PD participants was captured perceptually but not using automated system. Regarding healthy controls, human rater and computerized method agreed in 69 (92%) of non-hypomimic scores.

To estimate the number of affected facial areas we computed a cutoff for each facial marker whereby values above 95th percentile of control group were considered abnormal. Using the obtained cutoffs, we estimated that three or more facial areas were affected in 43 PD patients (47%) and in only 5 healthy subjects (7%) (Fig. 2B).The most commonly disrupted facial regions included mouth (52%), jaw (40%), forehead (38%), and nose root (38%) (Fig. 2C).

### Relationship between facial features and clinical and imaging parameters

We found significant correlations between the bradykinesia/rigidity composite score taken from the MDS-UPDRS and automatic video-based facial markers describing eyebrow elevation/depression (*r* = −0.25, *p* = 0.019), upper lip elevation/depression (*r* = −0.24, *p* = 0.026), lower lip elevation/depression (*r* = −0.37 *p* < 0.001), mouth corner adduction/abduction (*r* = −0.34, *p* = 0.001), and jaw elevation/depression (*r* = −0.31, *p* = 0.004). In addition, significant correlations were detected between the total MDS-UPDRS III and facial markers describing eyebrow elevation/depression (*r* = −0.22, *p* = 0.042), upper lip elevation/depression (*r* = −0.24, *p* = 0.025), lower lip elevation/depression (*r* = −0.35, *p* < 0.001), mouth corner adduction/abduction (*r* = −0.32, *p* = 0.003), and jaw elevation/depression (*r* = −0.28, *p* = 0.008). The relationship analysis did not reveal any significant correlation between automatic hypomimia assessment and postural instability/gait difficulty score (Supplementary Table 3).

The analysis of relationships between facial markers and DAT-SPECT markers for more affected side (see Supplementary Table 4 for analysis across less affected side and mean value of both sides) revealed significant correlations between caudate binding and lower lip elevation/depression (*r* = 0.23, *p* = 0.032) and lateral canthal lines (*r* = −0.27, *p* = 0.011), as well as between putamen binding ratio and upper lip elevation/depression (*r* = 0.23, *p* = 0.028), lower lip elevation/depression (*r* = 0.32, *p* = 0.002), mouth corner adduction/abduction (*r* = 0.29, *p* = 0.005), and jaw elevation/depression (*r* = 0.28, *p* = 0.008). No other relationships between facial dynamic markers and clinical manifestations (Supplementary Table 3) or brain imaging markers were found (Supplementary Table 4).

## Discussion

This is the first study to demonstrate a fully automated objective approach assessing facial expressivity of a large drug-naïve de-novo PD cohort, using the natural and unconstrained monologue video recordings. Using the proposed technology detecting hypomimia patterns in both upper and lower face areas, we were able to distinguish PD patients and controls with 78% accuracy. Therefore, the objective analysis of hypomimia may provide a novel biomarker in PD and other α-synuclein-related diseases. The intriguing potential of the facial analysis is that it is easily interpretable, inexpensive, and non-invasive, and video recordings can be made even from patients’ home facilitating future scalability to a larger population.

The results of our study confirmed hypomimia as a common manifestation in de-novo PD^3,4,6^, which is in accordance with previous research reporting impaired facial exression in 26–37% of de-novo PD^4,7^. The combination of hypomimia and hypokinetic dysarthria assessed using MDS-UPDRS III items 3.1 and 3.2 has been reported to be able to distinguish newly diagnosed PD from controls with AUC = 0.80^5^. Our results based solely on perceptual hypomimia assessment revealed mild to moderate/severe hypomimia in 45% of PD participants reaching AUC = 0.81 and confirming the high discriminative potential of facial expressivity assessment. In comparison to clinical assessment, our instrumental analysis found that 57% of PD patients manifested hypomimia, which led to a slightly higher discrimination accuracy of AUC = 0.87 between PD and controls.

The automatic assessment captured differences in nearly all the predefined regions of interest regardless of whether the markers were based on surface or geometrical properties. In a closer look, the first and the second most common manifestations were decreased variability of movements in the mouth and jaw, representing diminished movements of the lower face. Indeed, most of the lower face movements are performed voluntarily during the speech therefore, we may assume that in the freely spoken monologue, these two aspects reflect a large portion of deficit in the voluntary facial movements^8^. Finally, the third and the fourth most common detected deficiencies were a decreased variability in forehead and nose root wrinkles, which primarily represent a decrease in the spontaneous expressivity during the monologue^9^. A reduction in blinking rate and eyelid movements in general represented by a reduction in palpebral aperture variability, decreased amount of eyebrow elevation/depression, and diminished cheek muscle activity were also observed. Our findings thus suggest that hypomimia manifestations are homogeneously distributed across the entire facial area, and both voluntary and spontaneous type movements are affected. We did not find lateral asymmetry in expressivity across investigated facial areas, although a more detailed investigation of laterality in facial expressivity is warranted^10,11^.

In accordance with previously published results^4,7^, our findings confirmed the association between severity of overall motor performance and the hypomimia of the lower and the upper face. Regarding bradykinesia and rigidity, previous neurophysiological studies of voluntary facial dynamics reported reduced velocity and amplitude of movements and increased tone during repetitive syllable production resulting from bradykinesia of facial muscles^3,12^. Similarly, the previous literature reported an association between the rigidity of labial muscles, decrement of lip movements, and electromyographic activity of orbicularis oris and mentalis muscles^13^. Moreover, the recent neuroimaging study showed that PD patients with hypomimia display significantly lower DAT-SPECT specific binding ratios in the putamen and caudate, supporting the hypothesis of hypomimia sharing common pathophysiology with bradykinesia^13^. Indeed, we found a relationship between bradykinesia/rigidity composite sub-score and lower face hypomimia represented by the diminished movement of mouth lips and corners as well as diminished jaw movement, while our results did not confirm any relationship between hypomimia and postural instability/gait difficulty sub-score. This is further supported by results of DAT-SPECT analysis, which revealed a significant correlation between putamen binding ratio and markers describing mouth and jaw movements as well as between caudate binding ratio and lower lip movement. Together, this strong evidence suggests that hypomimia is mainly associated with striatal dopaminergic deficit related to appendicular involvement. We did not find the relation between hypomimia and the extent of depression or cognitive deficits, which is in line with previous studies^14,15^.

The strength of this study is that we focused on assessing hypomimia via freely spoken monologue, which represents the most natural and available source of facial movements. The assessment of freely spoken monologue holds the greatest potential as the most convenient hypomimia measurement, which could be easily implemented in video communication systems and therefore scalable to a large population^16^. In addition, the freely spoken monologue extends the assessment of deliberate facial movement by the involvement of spontaneous facial expressions as a constituent of nonverbal communication and therefore provides a comprehensive picture of the hypomimia impact. However, this is at the expense of fully separating voluntary and spontaneous events. Even though the lower face is likely more affected by voluntary speech movements while the upper face is by spontaneous facial expressions^8^, we cannot entirely prevent events like spontaneous smiling or deliberate raising of eyebrows. Therefore, the detailed analysis of different movement types is a matter of future research based on specialized mimic tasks. Yet, pilot studies aimed at an objective assessment of deliberate facial movement support the effect of PD on reduced speed and peak amplitude of the intentional facial grimacing^16–29^. On the other hand, the static assessment of facial shape provided only limited results supporting dynamic objective assessment as a superior approach^30^. Table with a detailed list of previously published literature is available in Supplementary Table 5.

One potential limitation of our method is that marker evaluating variability of lateral canthal lines was not able to capture a decrease in facial expressivity. This is surprising because the occurrence of lateral canthal lines is one of the main signs of Duchenne smile, involving the cheek raiser muscle^31^. In PD, the Duchenne smile is usually reduced, and smiling is produced only by the involvement of lower face muscles, causing an impression of disingenuity^32^. The insignificant lateral canthal lines marker might result from two issues, including occasional obscurement by hair or temples of participant’s glasses and/or the reduced visibility of lateral canthal areas due to its perpendicular orientation towards the image plane. This assumption is supported by significant impoverishment of the activity in the cheek area, which also reflects the orbicularis oculi activation during a Duchenne smile^33^. The second limitation is that our database was composed of only Czech native speakers of Slavic origin and was not tested on participants with different ethnic background. Nonetheless the face landmark tracking is based on the model trained on the 300 Faces-in-the-Wild database^34^, which contains representatives of different ethnicities and therefore the face tracking should yield similar results regardless of ethnic background. Moreover, the facial markers were based on relative changes in the facial movement which decreases effects of different ethnic attributes. Subsequently, patients with moderate level of depression were excluded to avoid potential negative effect of depression of facial expression^35^ that might led to overestimation of hypomimia prevalence in PD. On the other hand, we believe the presence of depression would not affect the algorithm reliability. Future research is warranted to explore effect of depression on facial expression in PD. Finally, to get as natural setup as possible, participant head position was not fixed during the freely spoken monologue and therefore, the measurements might be affected by head movements or lighting to a certain degree. To minimize this effect, patients were always positioned heading towards camera, which was placed in fixed position with regards of lighting. The Euclidean markers were normalized using distance of the inner eye corners and the surface markers were estimated from grayscale images with intensity levels normalized between zero and one and additionally, the markers with left and right variants were averaged for further analysis. The video frame sequence with obscured or out of frame landmark positions were not considered if they were longer than one second. From clinical point of view, we analyzed de-novo drug-naïve PD patients without levodopa-induced dyskinesias that would have potential to substantially affect the head position.

In conclusion, we introduced an automatic video-based assessment of hypomimia able to reliably capture distortion of various facial movement patterns, supporting the addition of automated facial assessment to the batteries of motor biomarkers currently used in clinical trials. Our results support an association between striatal dopaminergic deficit and hypomimia in PD. The presented method enables sufficiently sensitive and clinically relevant measurement of facial dynamic and opens the door for more intensive research of longitudinal evolution of hypomimia and its relationship with other disease symptoms as well as responsiveness to therapy. It will also provide a better understanding of the underlying mechanism for disrupted facial expressivity in PD and may ultimately lead to more efficient personalized hypomimia therapy or more accurate prediction of disease prognosis.

## Methods

### Standard protocol approvals, registrations, and patient consents

All participants provided written informed consent prior to their inclusion. Additionally, the person portrayed in Supplementary Video 1 provided written permission to publish the recording. The study received approval from the ethics committee of General University Hospital in Prague, Czechia, and has been performed in accordance with the ethical principles laid down by the Declaration of Helsinki.

### Study design and participants

From 2015 to 2020, we recruited de-novo, drug-naïve PD patients at the Department of Neurology, Charles University, and General University Hospital in Prague, Czechia for this cross-sectional study. Each patient was diagnosed according to Movement Disorder Society clinical diagnostic criteria for PD^36^ and underwent the clinical assessment including (i) structured interview gathering information about personal and medical history, history of drug substance intake, and current medication usage, (ii) semi-quantitative testing of PD motor symptoms with Movement Disorder Society-Unified Parkinson’s Disease Rating Scale, Part III^37^ (MDS-UPDRS III), (iii) cognitive testing with the Montreal Cognitive Assessment^38^ (MoCA), and (iv) evaluation of depressive symptoms with Beck Depression Inventory II^39^ (BDI II). Two sub-scores were calculated from the MDS-UPDRS III; bradykinesia/rigidity composite score was defined as the sum of items 3.3–3.8 and 3.14, and postural instability/gait difficulty (PIGD) sub-score was defined as the sum of items 3.9–3.13. Disease duration was estimated based on the self-reported occurrence of the first motor symptoms. In addition to the PD cohort, we examined the age- and the sex-matched control group of healthy participants. A neurologist experienced in movement disorders (P.D.) established PD diagnosis in all patients and performed all clinical evaluations.

The exclusion criteria were (i) history of therapy with antiparkinsonian medication, (ii) history of significant neurological disorders (except PD in the patient group), (iii) history of other clinical conditions affecting facial movements such as facial paralysis, hemifacial spasm, or stroke (vi) presence of moderate or severe depression defined as BDI II ≥ 20; (v) current or past involvement in any speech or hypomimia therapy.

### Dopamine transporter imaging

In PD patients, the dopamine transporter single-photon emission computed tomography (DAT-SPECT) was performed using the [123I]-2-b-carbomethoxy-3b-(4-iodophenyl)-N-(3-fluoropropyl) nortropane (DaTscan^®^, GE Healthcare) tracer according to the European Association of Nuclear Medicine procedure guidelines^40^, using common acquisition and reconstruction parameters described in detail previously^41^. Automated semi-quantitative analysis was performed using the BasGan V2 software^42^, and specific binding ratios in caudate nuclei and putamina relative to background binding were calculated. Since neurodegeneration of substantia nigra is frequently asymmetric in early PD, binding ratios in left and right side were calculated separately and the lower value from both hemispheres was used in subsequent analyses^43^.

### Facial expressivity examination

Facial expressivity examination was performed in a room with common indoor lighting with fixed position regarding artificial and natural sources of light, using video recordings obtained by the digital camera (Panasonic Handycam HDR-CX410, Osaka, Japan) placed approximately one meter in front of the subject’s face. The recording was performed with a resolution of 1440 × 1080 pixels (HD) and a frame rate of 25 RGB images (24-bit) per second. Each recording contained one minute of the freely spoken monologue on the given topic. The monologue was part of a comprehensive speech examination protocol performed by a speech specialist (M.N., T.T., or J.R.) during a single session.

### Computerized analysis of facial expressivity

During the computerized analysis, the state-of-the-art convolutional neural network localized sixty-eight facial landmarks in each video frame^44,45^. Using the detected facial landmarks, twelve hypomimia markers describing the dynamic of eight different facial regions were estimated (Supplementary Video 1). Due to the different nature of individual markers, two basic approaches were adopted: (i) Euclidean distance between facial landmarks and (ii) surface description of predefined areas of interest. If the left and right marker variants exist, both variants were estimated.

**Fig. 3:**
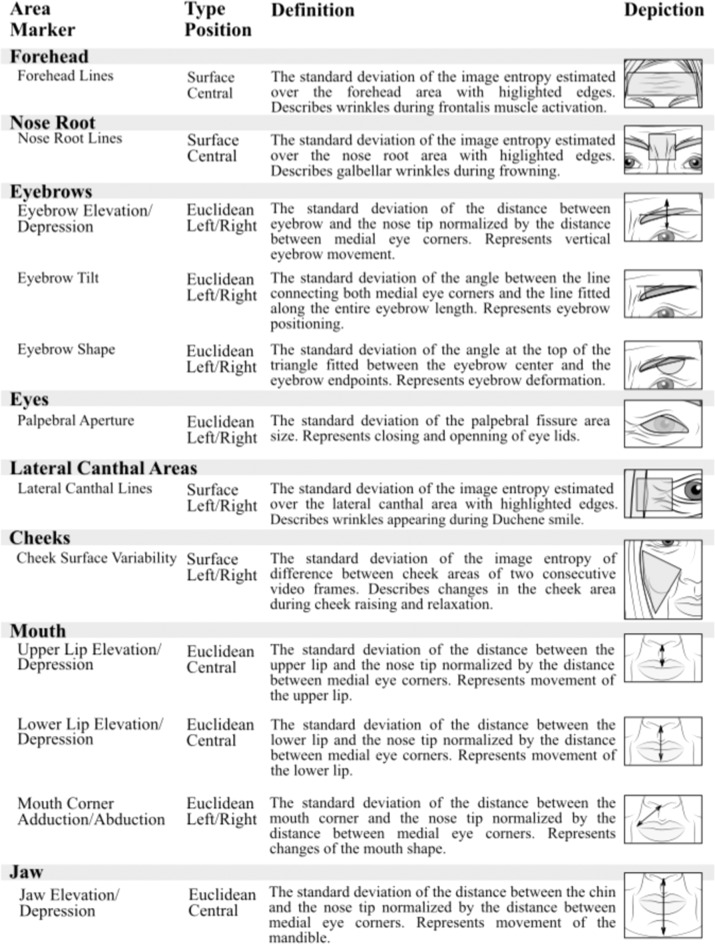
Illustration of analyzed facial areas. Detailed description of assessed markers of facial dynamics. All markers are expected to decrease in parallel with increasing severity of facial bradykinesia.

Based on the previous literature, regions of interest describing the entire facial area were selected^3^. The analyzed areas and the definitions of all facial markers are illustrated in Fig. 3. The eight described areas included forehead, nose root, eyebrows, *eyes*, lateral canthal areas, cheeks, mouth, and jaw. The forehead area was described by one surface marker centered along the vertical axis. The forehead lines, defined as the standard deviation of the image entropy estimated over the forehead area with highlighted edges, represented wrinkles created by the frontalis muscle activation. The nose root area was described by one surface marker centered along the vertical axis. The nose root lines, defined as the standard deviation of the image entropy estimated over the nose root area with highlighted edges, represented glabellar wrinkles created during frowning. The movements assigned to eyebrows area was described by three Euclidean markers with left/right variants. The eyebrow elevation/depression, defined as the standard deviation of the distance between eyebrow and the nose tip normalized by the distance between medial eye corners, represented vertical eyebrow movement. The eyebrow tilt, defined as the standard deviation of the angle between the line connecting both medial eye corners and the line fitted along the entire eyebrow length, represented eyebrow positioning. The eyebrow shape, defined as the standard deviation of the angle at the top of the triangle fitted between the eyebrow center and the eyebrow endpoints, represented eyebrow deformation. The area of eyes was described by one Euclidean marker with left/right variants. The *palpebral aperture*, defined as the standard deviation of the palpebral fissure area size, represented eye closing and opening. The lateral canthal areas were described by one surface marker with left/right variants. The *lateral canthal lines*, defined as the standard deviation of the image entropy estimated over the lateral canthal areas with highlighted edges, reflected lack of wrinkles in Duchenne smile, during which only lower face is involved without involvement of eyes and corresponding cheek raise. The cheek area was described by one surface marker with left/right variants. The *cheek surface variability*, defined as the standard deviation of the image entropy of difference between cheek areas of two consecutive video frames, represented changes in the cheek area during the raising and relaxation of cheeks. The mouth area was described by three Euclidean markers, two markers centered along the vertical axis and one with left/right variants. The *upper lip elevation/depression*, defined as the standard deviation of the distance between the upper lip and the nose tip normalized by the distance between medial eye corners, represents a movement of the upper lip. The *lower lip elevation/depression*, defined as the standard deviation of the distance between the lower lip and the nose tip normalized by the distance between medial eye corners, represented a movement of the lower lip. The *mouth corner adduction/abduction*, defined as the standard deviation of the distance between the mouth corner and the nose tip normalized by the distance between medial eye corners, represented changes in the mouth shape. Finally, the jaw area was described by one Euclidean marker centered along the vertical axis. The *jaw elevation/depression*, defined as the standard deviation of the distance between the chin and the nose tip normalized by the distance between medial eye corners, represented a movement of the mandible.

The landmark detection was performed in the Anaconda Individual Edition of Python 3.6 (Anaconda Inc., Austin, TX, USA). The subsequent dynamic marker assessment was conducted in MATLAB^®^ (Mathworks, Natick, MA, USA). A comprehensive description of the computerized hypomimia marker methodology can be found in Supplementary Information 1.

### Perceptual analysis of facial expressivity

The speech-language pathologist (H.R.) trained in facial expressivity evaluation provided an assessment of facial expressivity of each video recording assigning scores ranging from 0 for normal facial expressivity, 1 for slight hypomimia, 2 for mild hypomimia, and 3 for moderate/severe hypomimia. The rating criteria were anchored in the MDS-UPDSR III item 3.2 Facial Expression; the only exception was merged moderate/severe hypomimia score, as these two categories differ only by amount of time reflecting mouth at rest with parted lips, which is not possible to differentiate during monologue. The perceptual evaluation was performed on the randomized and blinded video recordings of monologues, including both PD and control group, while the rater could repeatedly replay video recording if needed.

### Statistical analysis

The one-sample Kolmogorov–Smirnov test was used to evaluate the normality of distributions. Group differences were calculated using analysis of variance for normally distributed data and the Kruskal–Wallis test for non-normally distributed data with the possible presence of outliers.

We addressed multiple comparisons via the Bonferroni adjustment for the nineteen comparisons, including twelve markers in which seven had left and right variants. The adjusted level of significance was set to *p* < 0.0026 (i.e., 0.05/19). Pearson’s partial correlation analysis controlled for age was used to test for associations between automatic video-based facial markers and the perceptual, clinical, and neuroimaging scales; the markers capturing the left- and right-side variant were represented by their average value.

To estimate the ability of markers of facial dynamic to distinguish between PD and control groups, we performed a binary logistic regression followed by leave-one-subject-out cross-validation. Several different classification scenarios were evaluated including classifiers based on single facial markers, and the combination yielding the best accuracy. The subset of facial markers providing the best accuracy was searched using a grid-search approach. The accuracy, sensitivity, and specificity values were computed to reflect the predictive value of dynamic facial markers. An overall indication of diagnostic accuracy was reported as the area under the curve (AUC), which we obtained from the receiver operating characteristic curve.

### Reporting summary

Further information on research design is available in the Nature Research Reporting Summary linked to this article.

## Supplementary information

Supplementary information 1.

Video 1. Facial markers

Written consent with publication of video material

Reporting Summary

## Data Availability

Individual participant data that underlie the findings of this study are available upon reasonable request from the corresponding author. The data are not publicly available due to their containing of information that could compromise the privacy of study participants.

## Code availability

The facial landmark detection was performed using a publicly available Python implementation of 2D Face Alignment Network^44^.
