# Supplementary material for: Automated video-based assessment of facial bradykinesia in de-novo Parkinson’s disease
Source: NPJ Digit Med. 2022 Jul 18;5:98. doi: 10.1038/s41746-022-00642-5 (PMC9293947; doi:10.1038/s41746-022-00642-5)
Supplement: Supplementary file 1 — Supplementary information 1. [file 41746_2022_642_MOESM1_ESM.docx]

**Supplementary Information 1:** Detailed description of the automatic evaluation of video

**Video processing**

At the beginning the original recordings were split into separate frames. Subsequently, each frame underwent analysis consisting of facial landmark detection, definition of facial regions of interest, image preprocessing ensuring the robustness of the analysis, and estimation of markers of facial dynamic.

**The facial landmark detection**

The facial landmark detection was performed on the original RGB images. It provided an estimate of 68 facial landmarks defining positions of eyebrows, eyes, nose, mouth, and outline of the jaw^1^ (Figure S1). A 2D-landmark model fitted by the convolutional neural network with 2D Face Alignment Network (FAN) architecture was used to determine the positions of the facial landmarks in each frame^2^. The FAN network employs a stack of four hourglass neural networks with bottleneck replaced by the hierarchical, parallel, and multiscale block, outperforming the previous bottleneck design^2^.


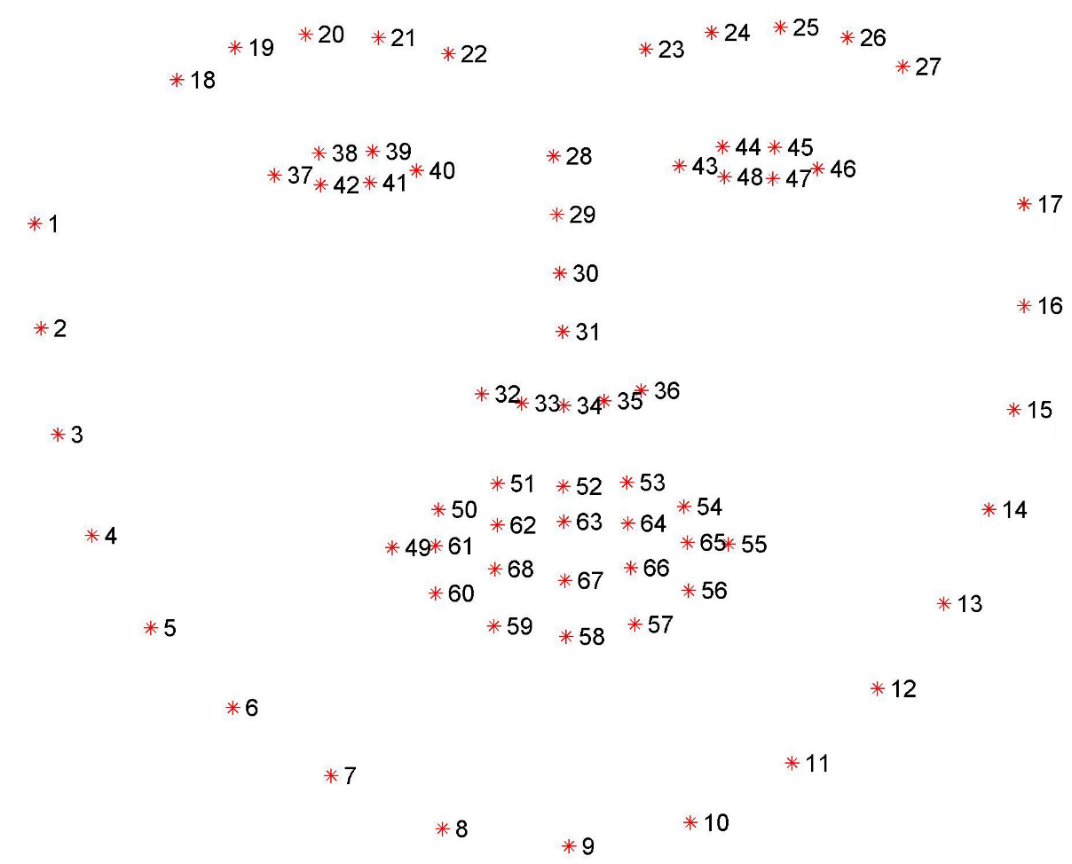


**Supplemetary Figure 1** 68 Facial landmarks of MultiPIE model^3^

In the first step, the FAN was pretrained on the 300-W-LP2D dataset of the synthetically generated dataset containing 61 225 faces with a large variety of poses^2^. The 300-W-LP2D was created by warping the original 2 000 manually annotated images of the 300-W dataset, currently the most used in-the-wild dataset in the field of facial landmark detection^2^. Finally, the FAN was fine-tuned on the original 300-W database containing 2 000 real images^2^ to alleviate discrepancies in the positions between synthetic and authentic images.

**The image preprocessing**

We have encountered two events during which the landmark detection failed (i) concealments of the participant's face and (ii) false surplus detections of the face. Regarding the robustness of the algorithm, both events had a profound effect on the performance.

The concealment issue was partially solved using the Kalman filter, which predicted the position of the concealed landmark based on its previous detections. Because we performed an offline analysis of previously recorded data, we were able to filter each signal from both directions (beginning-to-end and end-to-beginning) and combine filtered signals, which further enhanced the predictive value. The combination was performed as a sum of weighted signals, where the first set of weights $w_{1}$ was linearly increasing in the range between zero to one, with the one closest to the last known position and zero closest to the last unknown position. The second set of weights was defined as $w_{2}=1-w_{1}$. However, this solution provided a robust estimate only for the concealments shorter than one second. Therefore, the longer concealments were excluded, and if the total length of the excluded signal exceeded the 30s, the entire recording was discarded from further analysis.

Considering the false facial detections, the center of the video frame was filled with the participant's face. Thus, we were able to easily distinguish false detections using Euclidean distance from the reference facial landmark, which was the detected nose tip closest to the image center. In addition, the Hampel filter-based outlier detection was applied on the sum of Euclidean distances between the tip of the nose and all remaining 67 detected facial landmarks. The outlier detection excluded markedly deformed facial landmark models. A

To increase the performance of the facial markers based on surface properties, every image was transferred to grayscale. In addition, the value of each pixel was normalized between values zero to one.

**The estimation of markers of facial dynamic**

The marker descriptions utilize the landmark numbering presented in the Figure S1; the numbers of facial landmarks are reported in the brackets after the landmark of their reference. For the facial dynamic assessment, the two types Euclidean and surface markers were evaluated. The first eight markers were computed directly from Euclidean distances between the landmark positions and included movement markers of:

1. eyebrow elevation/depression,
2. eyebrow tilt,
3. eyebrow shape,
4. palpebral aperture,
5. upper lip elevation/depression,
6. lower lip elevation/depression,
7. mouth corner adduction/abduction, and
8. jaw elevation/depression.

The *eyebrow elevation/depression* was estimated in left right variants as a standard deviation in the height of eyebrow in all consecutive video frames. It was evaluated as median of all five eyebrow landmarks (18–22 or 23–27) relative to the distance of the nose tip landmark (31) and normalized by the distance of medial eye corners (40 or 43).

The *eyebrow tilt* was estimated in left/right variants, as the standard deviation of the angle between the line fitted to five eyebrow landmarks (18–22 or 23–27) and the line connecting medial eye corners (40 or 43) in all consecutive video frames.

The *eyebrow shape* was estimated in left/right variants as a standard deviation of the angle at the top of the triangle (20 or 25) between sides defined as lines between the eyebrow centers and endpoints (18–20 and 20–22, or 23–25 and 25–27) in all consecutive video frames.

The *palpebral aperture* was estimated in left/right variants as a standard deviation of the palpebral fissure area defined by six eye landmarks (37–42 or 43–48) in all consecutive video frames.

The *upper lip elevation/depression* was estimated in left/right variants as a standard deviation of the distance between the upper lip center (52) and the nose tip (31), normalized by the distance of medial eye corners (40 or 43) in all consecutive video frames.

The *lower lip elevation/depression* was estimated in left/right variants as a standard deviation of the distance between the lower lip center (58) and the nose tip (31), normalized by the distance of medial eye corners (40 or 43) in all consecutive video frames.

The *mouth corner adduction/abduction* was estimated in left/right variants as a standard deviation in the distance between mouth corners (49 or 55) and nose tip (31), normalized by the distance of medial eye corners (40 or 43) in all consecutive video frames.

The *jaw elevation/depression* was estimated as a standard deviation of distance between lowest jaw landmark (9) and nose tip (31) normalized the distance of medial eye corners (40 or 43) in all consecutive video frames.

The remaining four markers described surface properties of the predefined facial regions of interest, including

1. the forehead lines,
2. the nose root lines,
3. the lateral canthal lines, and
4. the cheek surface variability.

The *forehead lines* marker was estimated over an area defined by the intersections of four lines: two defined by the lateral landmarks of eyebrows (18 and 27) and perpendicular to the line of the nose (28 to 31). And two defined by the landmarks superior from the top of the eyebrows (20 or 25) in the distance of one-tenth, respectively one distance between nose bridge (28) and nose tip (31) and perpendicular to the line connecting medial eye corners (40 and 43). The selected region of interest was reshaped to a rectangular shape using a homomorphic transformation and resized to the width of 250 pixels with the preserved aspect ratio. The forehead marker was estimated using standard deviation of the sums of the range filtered images in all consecutive video frames.

The *nose root lines* marker was estimated over an area defined by the intersections of four lines: two defined by the medial landmarks of eyebrows (21 and 23) and perpendicular to the line of the nose (28 to 31). The third defined by the landmark locating the inferior end of the nose bridge (28) and perpendicular to the line connecting medial eye corners (40 and 43) and the fourth perpendicular to the line connecting medial eye corners (40 and 43) intersecting point in the 1.5 distance between the beginning of the nose bridge (28) and the line connecting medial eyebrow ends (22 and 23). The selected region of interest was reshaped to a rectangular shape using a homomorphic transformation and resized to a height of 75 pixels with the preserved aspect ratio. The nose root marker was estimated using standard deviation of the sums of the range filtered images in all consecutive video frames.

The *lateral canthal lines* marker was estimated over an area defined by the intersections of four lines: the first defined by lateral eye corner (37 or 46) and perpendicular to nose line (28 to 31), the second its image mirrored over a line perpendicular to nose line (28 to 31) and intersecting lateral eyebrow (18 or 27), the third defined by a lateral eyebrow (18 or 27) end and perpendicular to the line connecting medial eye corners (40 and 43), and the fourth its image mirrored over the line perpendicular to the line connecting medial eye corners (40 and 43) and intersecting lateral eye corner (37 or 46). The selected region of interest was reshaped to a rectangular shape using a homomorphic transformation and resized to the width of 100 pixels with the preserved aspect ratio. The lateral canthal lines marker was estimated as a standard deviation in the sums of range filtered images in all consecutive video frames.

The *cheek surface variability* marker was estimated over an area defined by the connection of four landmarks: the side of the nose (32 or 36), the highest landmark of jawline (1 or 17), the fourth-highest landmark of the jawline (4 or 14), and the sixth-highest landmark of the jawline(6 or 12). The regions of interest of two consecutive images defined by these four landmarks were resized to 100-to-100-pixel squares, registered, and subsequently subtracted. To avoid the noise at the edges, the images were further cropped by 10 pixels, and the entropy of the cropped difference was used as a metric of the dynamic changes in the cheek regions. The cheek marker was estimated for the left and the right side of the face as a standard deviation of the entropy sums of all image differences across all videoframes.

**Supplementary References**

1. Bulat A, Tzimiropoulos G. How far are we from solving the 2d & 3d face alignment problem? (and a dataset of 230,000 3d facial landmarks). In: Proceedings of the IEEE International Conference on Computer Vision. 2017; pp 1021–1030. doi: 10.1109/ICCV.2017.116
2. Sagonas C, Antonakos E, Tzimiropoulos G, Zafeiriou S, Pantic M. 300 faces in-the-wild challenge: Database and results. Image Vis Comput 2016; 47: 3–18. doi: 10.1016/j.imavis.2016.01.002.
3. Gross R, Matthews I, Cohn J, Kanade T, Baker S. Multi-PIE. Proc Int Conf Autom Face Gesture Recognit. 2010 May 1;28(5):807-813. doi: 10.1016/j.imavis.2009.08.002.

**Video captions**

**Supplementary Video 1**. Video depicting facial landmark detection and the subsequent definition of the Euclidean facial markers and the regions of interest for the facial surface analysis.

**Supplementary Table 1.** Relationships between computerized markers and perceptual rankings. Bold numbers indicate significant values (*p* < 0.05).

| **Facial Marker** | **PD & controls** | |  | **PD** | |  | **controls** | |
| --- | --- | --- | --- | --- | --- | --- | --- | --- |
|  | **r** | **P** |  | **r** | **P** |  | **r** | **p** |
| Forehead | | | | | | | | |
| Forehead Lines | **-0.47** | **< 0.001** |  | **-0.29** | **< 0.01** |  | **-0.38** | **< 0.01** |
| Nose Root | | | | | | | | |
| Nose Root Lines | **-0.50** | **< 0.001** |  | **-0.23** | **< 0.05** |  | **-0.36** | **< 0.01** |
| Eyebrows | | | | | | | | |
| Eyebrow Elevation/Depression | **-0.54** | **< 0.001** |  | **-0.43** | **< 0.001** |  | **-0.29** | **< 0.05** |
| Eyebrow Tilt | -0.03 | = 0.70 |  | 0.16 | = 0.13 |  | -0.14 | = 0 .24 |
| Eyebrow Shape | **-0.44** | **< 0.001** |  | **-0.22** | **< 0.05** |  | **-0.33** | **< 0.01** |
| Eyes | | | | | | | | |
| Palpebral Aperture | **-0.38** | **< 0.001** |  | -0.17 | = 0.12 |  | **-0.30** | **< 0.01** |
| Lateral Canthal Areas | | | | | | | | |
| Lateral Canthal Lines | **-0.26** | **< 0.001** |  | -0.18 | = 0.10 |  | -0.23 | p = 0.05 |
| Cheeks | | | | | | | | |
| Cheek Surface Variability | -0.14 | = 0.08 |  | 0.02 | = 0.85 |  | -0.06 | =0.60 |
| Mouth | | | | | | | | |
| Upper Lip Elevation/Depression | **-0.51** | **< 0.001** |  | **-0.38** | **< 0.001** |  | **-0.30** | **< 0.01** |
| Lower Lip Elevation/Depression | **-0.47** | **< 0.001** |  | **-0.40** | **< 0.001** |  | -0.20 | = 0.09 |
| Mouth Corner Adduction/Abduction | **-0.53** | **< 0.001** |  | **-0.39** | **< 0.001** |  | **-0.30** | **< 0.01** |
| Jaw | | | | | | | | |
| Jaw Elevation/Depression | **-0.49** | **< 0.001** |  | **-0.40** | **< 0.001** |  | -0.21 | = 0.08 |

**Captions:** PD = Parkinson’s disease.

**Supplementary Table 2.** Results of hypomimia diagnostic sensitivity analysis listing AUC, accuracy sensitivity and specificity for single automatic facial markers.

| **Facial Marker** | **AUC** |  | | **Accuracy** | |  | **Sensitivity** |  | **Specificity** |
| --- | --- | --- | --- | --- | --- | --- | --- | --- | --- |
| Forehead | | | | | | | | | |
| Forehead Lines | 0.81 | |  | | 0.75 |  | 0.78 |  | 0.74 |
| Nose Root | | | | | | | | | |
| Nose Root Lines | 0.74 | |  | | 0.66 |  | 0.69 |  | 0.65 |
| Eyebrows | | | | | | | | | |
| Eyebrow Elevation/Depression | 0.78 | |  | | 0.71 |  | 0.72 |  | 0.70 |
| Eyebrow Tilt | 0.57 | |  | | 0.53 |  | 0.44 |  | 0.55 |
| Eyebrow Shape | 0.74 | |  | | 0.69 |  | 0.71 |  | 0.68 |
| Eyes | | | | | | | | | |
| Palpebral Aperture | 0.64 | |  | | 0.60 |  | 0.61 |  | 0.59 |
| Lateral Canthal Areas | | | | | | | | | |
| Lateral Canthal Lines | 0.58 | |  | | 0.56 |  | 0.53 |  | 0.57 |
| Cheeks | | | | | | | | | |
| Cheek Surface Variability | 0.64 | |  | | 0.60 |  | 0.60 |  | 0.60 |
| Mouth | | | | | | | | | |
| Upper Lip Elevation/Depression | 0.76 | |  | | 0.68 |  | 0.70 |  | 0.67 |
| Lower Lip Elevation/Depression | 0.77 | |  | | 0.69 |  | 0.68 |  | 0.69 |
| Mouth Corner Adduction/Abduction | 0.81 | |  | | 0.70 |  | 0.71 |  | 0.70 |
|  | | | | | | | | | |
| Jaw Elevation/Depression | 0.80 | |  | | 0.71 |  | 0.73 |  | 0.70 |

**Captions:** AUC = Area under curve.

**Supplementary Table 3.** Partial (age-adjusted) correlation analysis between facial markers and clinical parameters in PD. Bold numbers indicate significant values (*p* < 0.05).

| **Facial Marker** | **MDS-UPDRS III Total** | |  | **Bradykinesia /Rigidity** | |  | **PIGD** | |  | **MoCA** | |  | **BDI II** | |
| --- | --- | --- | --- | --- | --- | --- | --- | --- | --- | --- | --- | --- | --- | --- |
|  | **r** | **p** |  | **r** | **p** |  | **r** | **p** |  | **r** | **p** |  | **r** | **p** |
| Forehead | | | | | | | | | | | | | | |
| Forehead Lines | -0.10 | = 0.34 |  | -0.13 | = 0.21 |  | 0.00 | = 0.97 |  | 0.10 | = 0.29 |  | 0.07 | = 0.47 |
| Nose Root | | | | | | | | | | | | | | |
| Nose Root Lines | -0.10 | = 0.38 |  | -0.13 | = 0.24 |  | -0.16 | = 0.13 |  | 0.00 | = 0.96 |  | -0.15 | = 0.19 |
| Eyebrows | | | | | | | | | | | | | | |
| Eyebrow Elevation/Depression | **-0.22** | **=0.042** |  | **-0.25** | **= 0.019** |  | -0.14 | = 0.18 |  | 0.00 | = 0.97 |  | -0.08 | = 0.45 |
| Eyebrow Tilt | 0.11 | = 0.30 |  | 0.05 | = 0.56 |  | 0.13 | = 0.22 |  | 0.08 | = 0.47 |  | 0.00 | = 0.97 |
| Eyebrow Shape | -0.11 | = 0.30 |  | -0.12 | = 0.25 |  | -0.03 | = 0.77 |  | 0.09 | = 0.41 |  | -0.12 | = 0.25 |
| Eyes | | | | | | | | | | | | | | |
| Palpebral Aperture | 0.10 | = 0.34 |  | 0.05 | = 0.62 |  | -0.02 | = 0.86 |  | -0.04 | = 0.69 |  | 0.11 | = 0.29 |
| Latheral Canthal Areas | | | | | | | | | | | | | | |
| Lateral Canthal Lines | **-**0.03 | = 0.76 |  | -0.02 | 0.89 |  | 0.05 | = 0.62 |  | -0.08 | = 0.48 |  | -0.08 | = 0.44 |
| Cheeks | | | | | | | | | | | | | | |
| Cheek Surface Variability | 0.08 | = 0.46 |  | 0.10 | = 0.33 |  | 0.09 | = 0.40 |  | -0.04 | = 0.72 |  | 0.04 | = 0.74 |

| Mouth | | | | | | | | | | | | | | |
| --- | --- | --- | --- | --- | --- | --- | --- | --- | --- | --- | --- | --- | --- | --- |
| Uper Lip Elevation/Depression | **-0.24** | **= 0.025** |  | **-0.24** | **= 0.026** |  | -0.05 | = 0.68 |  | 0.12 | = 0.29 |  | 0.07 | = 0.53 |
| Lower Lip Elevation/Depression | **-0.35** | **< 0.001** |  | **-0.37** | **< 0.001** |  | -0.12 | = 0.27 |  | 0.07 | = 0.53 |  | -0.06 | = 0.57 |
| Mouth Corner Adduction/Abduction | **-0.32** | **= 0.003** |  | **-0.34** | **= 0.001** |  | -0.12 | = 0.26 |  | 0.09 | = 0.38 |  | -0.10 | = 0.35 |
| Jaw | | | | | | | | | | | | | | |
| Jaw Elevation/Depression | **-0.28** | **= 0.008** |  | **-0.31** | **= 0.004** |  | -0.13 | = 0.25 |  | 0.08 | = 0.48 |  | -0.04 | = 0.68 |

**Captions:** PD = Parkinson’s disease; MDS-UPDRS = Movement Disorders Society – Unified Parkinson’s Disease Rating Scale; PIGD = postural instability/gait difficulty; MoCA = Montreal Cognitive Assessment; BDI II = Beck Depression Inventory II.

**Supplementary Table 4.** Partial (age-adjusted) correlation analysis between facial markers and imaging parameters in PD. Bold numbers indicate significant values (*p* < 0.05).

| **Facial Marker** | **Caudate binding ratio** | | | | | | | |  | **Putamen binding ratio** | | | | | | | |
| --- | --- | --- | --- | --- | --- | --- | --- | --- | --- | --- | --- | --- | --- | --- | --- | --- | --- |
|  | **More affected side** | |  | **Less affected side** | |  | **Mean value** | |  | **More affected side** | |  | **Less affected side** | |  | **Mean value** | |
|  | **r** | **p** |  | **r** | **p** |  | **r** | **p** |  | **r** | **p** |  | **r** | **p** |  | **r** | **p** |
| Forehead | | | | | | | | | | | | | | | | | |
| Forehead Lines | 0.14 | = 0.19 |  | 0.10 | = 0.37 |  | 0.12 | = 0.26 |  | 0.15 | = 0.15 |  | 0.15 | = 0.16 |  | 0.16 | = 0.13 |
| Nose Root | | | | | | | | | | | | | | | | | |
| Nose Root Lines | 0.14 | = 0.20 |  | **0.28** | **= 0.008** |  | **0.22** | = **0.038** |  | 0.17 | = 0.11 |  | **0.39** | **= 0.001** |  | **0.32** | **= 0.002** |
| Eyebrows | | | | | | | | | | | | | | | | | |
| Eyebrow Elevation/Depression | 0.15 | = 0.16 |  | 0.09 | = 0.42 |  | 0.12 | = 0.27 |  | 0.17 | = 0.10 |  | 0.14 | = 0.21 |  | 0.16 | = 0.13 |
| Eyebrow Tilt | -0.02 | = 0.83 |  | 0.06 | = 0.57 |  | 0.02 | = 0.83 |  | 0.04 | = 0.73 |  | 0.13 | = 0.23 |  | 0.10 | = 0.34 |
| Eyebrow Shape | -0.05 | = 0.64 |  | -0.05 | = 0.63 |  | -0.06 | = 0.57 |  | 0.11 | = 0.31 |  | 0.04 | = 0.70 |  | 0.06 | = 0.58 |
| Eyes | | | | | | | | | | | | | | | | | |
| Palpebral Aperture | 0.14 | = 0.20 |  | 0.10 | = 0.34 |  | 0.12 | = 0.26 |  | -0.03 | = 0.79 |  | 0.05 | = 0.64 |  | 0.02 | = 0.83 |
| Latheral Canthal Areas | | | | | | | | | | | | | | | | | |
| Lateral Canthal Lines | **-0.27** | **= 0.011** |  | -0.17 | = 0.11 |  | **-0.24** | = **0.027** |  | -0.09 | = 0.40 |  | 0.08 | = 0.44 |  | -0.00 | = 0.96 |
| Cheeks | | | | | | | | | | | | | | | | | |
| Cheek Surface Variability | 0.14 | = 0.18 |  | 0.12 | = 0.29 |  | 0.13 | = 0.21 |  | -0.04 | = 0.70 |  | -0.01 | = 0.96 |  | -0.01 | = 0.91 |

| Mouth | | | | | | | | | | | | | | | | | |
| --- | --- | --- | --- | --- | --- | --- | --- | --- | --- | --- | --- | --- | --- | --- | --- | --- | --- |
| Uper Lip Elevation/Depression | 0.15 | = 0.17 |  | 0.20 | = 0.06 |  | 0.18 | = 0.09 |  | **0.23** | **= 0.028** |  | **0.22** | **= 0.035** |  | **0.25** | = **0.019** |
| Lower Lip Elevation/Depression | **0.23** | **= 0.032** |  | 0.18 | = 0.09 |  | **0.21** | = **0.049** |  | **0.32** | **= 0.002** |  | 0.18 | = 0.09 |  | **0.26** | = **0.016** |
| Mouth Corner Adduction/Abduction | 0.12 | = 0.25 |  | 0.12 | = 0.28 |  | 0.12 | = 0.26 |  | **0.29** | **= 0.005** |  | **0.22** | **= 0.044** |  | **0.26** | = **0.014** |
| Jaw | | | | | | | | | | | | | | | | | |
| Jaw Elevation/Depression | 0.13 | = 0.22 |  | 0.14 | = 0.18 |  | 0.14 | = 0.19 |  | **0.28** | **= 0.008** |  | 0.19 | = 0.07 |  | **0.25** | = **0.020** |

**Captions:** PD = Parkinson’s disease.

**Supplementary Table 5.** Automatic video-based approaches for PD-related hypomimia assessment.

|  | **Objectives** | **Recording settings** | **Recorded task** | **Cohort** | **Disease severity** | **Disease duration (years)** | **Technology** | **Outcomes** |
| --- | --- | --- | --- | --- | --- | --- | --- | --- |
| *Yang et al. (2022)* | | | | | | | | |
|  | Hypomimia description | Video recordings | Sustained phonation | 16 PD (confirmed, medication: ON) & 16 HC | UPDRS III:  35.44 ± 15.62,  H&Y:  2.44 ± 0.68 | 4.79 ± 4.62 | Noldus FaceReader 7.0 - facial emotion recognition | Significant differences between all examined facial expressions. |
| *Abrami et al. (2021)* | | | | | | | | |
|  | Binary detection PD/HC and ON/OFF drug state | Web-based video recordings | Monologue | 107 PD (self-declared, medication: NA) & 1595 HC,  35 PD (confirmed, medication: ON & OFF),  Alan Alda interviews prior and post-diagnosis | Examined dataset (35 PD)  UPDRS III:  25± 13 | NA | Deep neural network pre-trained on healthy cohort | AUROC > 0.7,  ACC =70% |
| *Ali et al. (2021)* | | | | | | | | |
|  | Binary detection of PD/HC | Web-based video recordings | Posed grimacing | 61 PD (confirmed, medication: NA) & 543 HC | NA | 8.36 ±5.74 | AU analysis in combination with SVM | AUROC = 0.94,  ACC = 95.6 %,  F1 = 95% |
| *Gomez-Gomez et al. (2021)* | | | | | | | | |
|  | Binary detection of PD/HC | Video recordings | Evoked and posed grimacing | 30 PD (confirmed, medication: NA) & 24 HC | MDS-UPDRS III: 33.1 ± 13.3,  H&Y:  2.4 ± 0.5 | 11.46 ±10.16 | Multimodal PD detection and AU analysis | ACC= 88.5% |
| *Jakubowski et al. (2021)* | | | | | | | | |
|  | Binary detection of PD/HC | Video recordings  and thermal camera recordings | Posed grimacing | 24 PD (confirmed,] medication: NA) & 24 HC | NA |  | Facial features extracted by CNN in combination with SVM | F1 = 94.1 % |
| *Su et al.(2021a)* | | | | | | | | |
|  | Binary detection of PD/HC and hypomimia description | Video recordings | Posed grimacing | 47 PD (medication: NA) & 39 HC | NA | NA | Semantic Feature neural network | ACC= 94.2 %,  F1= 95.3 % |
| *Su et al.(2021b)* | | | | | | | | |
|  | Binary detection of PD/HC | Video recordings | Posed grimacing | 47 PD (medication: NA) & 39 HC | NA | NA | Geometric and Texture features in combination with SVM | F1 = 99.97 % |
| *Skibińska and Burget (2020)* | | | | | | | | |
|  | Binary detection of PD/HC | Video recordings | Sentence repetition | 70 PD (confirmed, medication: NA) & 45 HC | UPDRS III:  24.5 ± 12.1 | 7.6 ± 4.7 | Facial emotion recognizer | ACC = 69 % |
| *Grammatikopoulou et al. (2019)* | | | | | | | | |
|  | Hypomimia description | Video recordings | Selfie | 221 PD (self-declared),  23 PD (confirmed, medication: NA) & 11 HC | Examined dataset (23 PD) UPDRS Part III Item 19:  2.0 ± 0.7 | NA | iPrognosis cloud assessment of facial features | SENS = 0.79,  SPEC = 0.82 |
| *Rajnoha et al. (2018)* | | | | | | | | |
|  | Binary detection of PD/HC | Static face image | - | 50 PD (confirmed, medication: ON) & 50 HC | UPDRS III: 21.5±12.3 | 7.2 ± 3.4 | Geometrical features generated by CNN in combination with RF classifier | ACC = 67.3% |
| *Joshi et al. (2018)* | | | | | | | | |
|  | Hypomimia severity mapping on 5-point Likert scale | Video recordings | Monologue | 117 PD (confirmed, medication: NA) | NA | NA | AU in combination with Hierarchical Bayesian Neural Network | F1 = 0.50,  MAE = 0.49 |
| *Bandini et al. (2017)* | | | | | | | | |
|  | Hypomimia description | Video recordings | Posed grimacing | 17 PD (confirmed, medication: ON) & 17 HC | UPDRS III:  17.2 ± 10.1,  H&Y:  2.1 ± 0.4 | 7.82 ± 5.07 | Euclidean distance between posed facial expressions and neutral faces | Significantly more pronounced distances between emotional and neutral faces in the HC group |
| *Joshi et al. (2016)* | | | | | | | | |
|  | Hypomimia severity mapping on 5-point Likert scale | Video recordings | Monologue | 117 PD (confirmed, medication: NA) | NA | NA | Geometrical features in combination with RF classifier | MAE = 0.56 |
| *Vinokurov et al. (2015)* | | | | | | | | |
|  | Binary detection of PD/HC and hypomimia description | Depth camera recordings | Evoked grimacing | 14 PD (confirmed, medication: NA) & 15 HC | NA | NA | Faceshift facial feature detection in combination with linear regression | AUROC = [0.9-0.99] |

**Captions:** PD = Parkinson’s disease, HC = Healthy controls, UPDRS = Unified Parkinson’s Disease Rating Scale, MDS-UPDRS = Movement Disorders Society – Unified Parkinson’s Disease Rating Scale, H&Y = Hoehn and Yahr, AU = Action units, SVM = Support vector machine, CNN = Convolutional neural network, RF = Random forest, AUROC = Area under receiver operating curve, ACC = Accuracy, F1 = F1 score, SENS = Sensitivity, SPEC = Specificity, MAE = Mean average error, NA= Not available.
