# Supplementary material for: Automated video-based assessment of facial bradykinesia in de-novo Parkinson’s disease
Source: NPJ Digit Med. 2022 Jul 18;5:98. doi: 10.1038/s41746-022-00642-5 (PMC9293947; doi:10.1038/s41746-022-00642-5)
Supplement: Supplementary file 3 — Written consent with publication of video material [file 41746_2022_642_MOESM3_ESM.docx]

I hereby declare that I grant permission to Nature Publishing Group, a division of Macmillan Publisher Ltd, to publish the Supplementary Video S1 and related images together with article “Automated video-based assessment of facial bradykinesia in de-novo Parkinson's disease,” featuring recording of my speech in all formats (i.e. print and digital) under an Open Access Creative Commons license.

Prague 22.09.2016

Michal Novotný

(person speaking in the video)

On behalf of the research team: Michal Novotný

(corresponding author)
