# Supplementary material for: Automated video-based assessment of facial bradykinesia in de-novo Parkinson’s disease
Source: NPJ Digit Med. 2022 Jul 18;5:98. doi: 10.1038/s41746-022-00642-5 (PMC9293947; doi:10.1038/s41746-022-00642-5)
Supplement: Supplementary file 4 — Reporting Summary [file 41746_2022_642_MOESM4_ESM.pdf]

nature portfolio

Corresponding author(s): Michal Novotny

Last updated by author(s): May 4, 2022

Reporting Summary

Nature Portfolio wishes to improve the reproducibility of the work that we publish. This form provides structure for consistency and transparency in reporting. For further information on Nature Portfolio policies, see our Editorial Policies and the Editorial Policy Checklist.

Statistics

For all statistical analyses, confirm that the following items are present in the figure legend, table legend, main text, or Methods section.

n/a	Confirmed	
☐	☒	The exact sample size (*n*) for each experimental group/condition, given as a discrete number and unit of measurement
☐	☒	A statement on whether measurements were taken from distinct samples or whether the same sample was measured repeatedly
☐	☒	The statistical test(s) used AND whether they are one- or two-sided<br>*Only common tests should be described solely by name; describe more complex techniques in the Methods section.*
☐	☒	A description of all covariates tested
☐	☒	A description of any assumptions or corrections, such as tests of normality and adjustment for multiple comparisons
☐	☒	A full description of the statistical parameters including central tendency (e.g. means) or other basic estimates (e.g. regression coefficient) AND variation (e.g. standard deviation) or associated estimates of uncertainty (e.g. confidence intervals)
☐	☒	For null hypothesis testing, the test statistic (e.g. *F*, *t*, *r*) with confidence intervals, effect sizes, degrees of freedom and *P* value noted<br>*Give P values as exact values whenever suitable.*
☒	☐	For Bayesian analysis, information on the choice of priors and Markov chain Monte Carlo settings
☒	☐	For hierarchical and complex designs, identification of the appropriate level for tests and full reporting of outcomes
☐	☒	Estimates of effect sizes (e.g. Cohen's *d*, Pearson's *r*), indicating how they were calculated

Our web collection on statistics for biologists contains articles on many of the points above.

Software and code

Policy information about availability of computer code

Data collection	No software was used.
Data analysis	The facial landmark detection was performed in Anaconda distribution of python 3.8 (Anaconda Inc., Austin, TX) and all the subsequent analyses were performed in MATLAB® (MathWorks, Natick, MA).

For manuscripts utilizing custom algorithms or software that are central to the research but not yet described in published literature, software must be made available to editors and reviewers. We strongly encourage code deposition in a community repository (e.g. GitHub). See the Nature Portfolio guidelines for submitting code & software for further information.

Data

Policy information about availability of data

All manuscripts must include a data availability statement. This statement should provide the following information, where applicable:

- Accession codes, unique identifiers, or web links for publicly available datasets
- A description of any restrictions on data availability
- For clinical datasets or third party data, please ensure that the statement adheres to our policy

Individual participant data that underlie the findings of this study are available upon reasonable request from the corresponding author. The raw recordings are not publicly available due to their contain of information that could compromise the privacy of study participants.

Field-specific reporting

Please select the one below that is the best fit for your research. If you are not sure, read the appropriate sections before making your selection.

☐ Life sciences ☒ Behavioural & social sciences ☐ Ecological, evolutionary & environmental sciences

For a reference copy of the document with all sections, see nature.com/documents/nr-reporting-summary-flat.pdf

Behavioural & social sciences study design

All studies must disclose on these points even when the disclosure is negative.

Study description	Quantitative cross-sectional study.
Research sample	This study is based on a large sample of 91 patients (54 men, 37 women) with mean age of 61.0 (standard deviation 12.3, range 34-81) years, only de-novo treatment-naïve Parkinson's disease was considered. The healthy control group consisted of 75 subjects (45 men, 30 women) of comparable age (mean 60.8, standard deviation 8.8, range 45–86 years) with no history of neurological or other clinical conditions affecting facial movements.
Sampling strategy	An ad-hoc power analysis based on one-way analysis of variance with GROUP as covariate indicated a recommended minimum overall sample size of 80 for 2 groups (i.e., a minimum sample size of 40 per one group), given expected large effect size (Cohen's f of 0.4) with the error probability α set at 0.05 and a false negative rate β set at 0.2 (i.e., power of 0.8). We included sample size of 166 subjects for 2 groups.
Data collection	Facial expressivity examination was performed in a room with common indoor lighting, using video recordings obtained by the digital camera (Panasonic Handycam HDR-CX410, Osaka, Japan) placed approximately one meter in front of the subject's face. The recording was performed with a resolution of 1440 x 1080 pixels (HD) and a frame rate of 25 RGB images (24-bit) per second. Each recording contained one minute of the freely spoken monologue on the given topic. The monologue was part of a comprehensive speech examination protocol performed by a speech specialist (M.N., T.T., or J.R.) during a single session.
Timing	From 2015 to 2020.
Data exclusions	A total number of 97 PD participants were examined, and six were subsequently excluded: one because the diagnosis was updated to corticobasal degeneration, and five had moderate depression level according to BDI II. No HC data were excluded from the analysis.
Non-participation	No participants fullfilling inclusion/exlusion criteria stated within manuscript dropped from the analyses.
Randomization	The computerized methods were based on fully automated and objective approach. The perceptual analysis was based on the aseessment of speech-language pathologist (H.R.) trained in facial expressivity who evaluated each video recording.

Reporting for specific materials, systems and methods

We require information from authors about some types of materials, experimental systems and methods used in many studies. Here, indicate whether each material, system or method listed is relevant to your study. If you are not sure if a list item applies to your research, read the appropriate section before selecting a response.

Materials & experimental systems

n/a	Involved in the study	
☒	☐	Antibodies
☒	☐	Eukaryotic cell lines
☒	☐	Palaeontology and archaeology
☒	☐	Animals and other organisms
☐	☒	Human research participants
☒	☐	Clinical data
☒	☐	Dual use research of concern

Methods

n/a	Involved in the study	
☒	☐	ChIP-seq
☒	☐	Flow cytometry
☒	☐	MRI-based neuroimaging

Human research participants

Policy information about studies involving human research participants

Population characteristics	See above.
Recruitment	The Parkinson's disease patients were recruited at General University Hospital in Prague. The control subjects were recruited from the general community through advertisements. No selection bias was present.

Ethics oversight	The study was approved by the Ethics Committee of the General University Hospital in Prague, Czech Republic and have therefore been performed in accordance with the ethical standards laid down in the 1964 Declaration of Helsinki and its later amendments. All participants provided written, informed consent to the neurological examination and recording procedure.

Note that full information on the approval of the study protocol must also be provided in the manuscript.
